# Very poor practices regarding breast cancer screening among Sudanese female workers at a secondary-level hospital: a cross-sectional study

**DOI:** 10.11604/pamj.2022.41.43.30179

**Published:** 2022-01-17

**Authors:** Hanaa Ibrahiem Altirifi, Osama Mohamed Elsanousi, Shahinaz Bedri

**Affiliations:** 1 Department of Nursing Faculty of Medicine and Health Science, University of Blue Nile, Ad-Damazin Sudan, Khartoum, Sudan,; 2 Department of Surgery, Ribat University Hospital, The National Ribat University, Khartoum, Sudan,; 3 Pathology Unit, School of Medicine, Ahfad University for Women, Khartoum, Sudan

**Keywords:** Cross-sectional studies, breast self-examination, breast neoplasms, delayed diagnosis, incidence, Sudan, early detection, cancer, mass screening, breast, health personnel

## Abstract

**Introduction:**

breast cancer (BC) mortality and morbidity burden in African countries is higher compared to western countries due to late diagnosis produced by deficient screening. We aimed to assess the level of knowledge, attitude and practice regarding breast cancer screening among Sudanese female workers at a secondary-level hospital.

**Methods:**

this is a cross-sectional study carried out at the largest governmental hospital of Ad-Damazin City (capital of Blue Nile State, south-eastern Sudan) in 2018. It surveyed female healthcare providers “group A” compared to the non-medical female staff at the same hospital “group B” to assess their awareness, beliefs and behavior concerning Breast Cancer Screening (BCS). Chi-squared and Student t-tests were used for analysis with a significant p value of <0.05.

**Results:**

participants were 110, included 78 (70.9%), (“group A”) and 32 (29.1%) (“group B”) women. Good overall knowledge score (47.4%) vs (43.8%), for “group A” and “group B”, respectively, p=0.000. Positive attitude was scored by 63 (80.8%) vs. 23 (71.9%) participants in “group A” and “group B” respectively, p= 0.305. Obvious denial trend regarding susceptibility to this disease was noted in both groups. BCS practices were seriously unsatisfactory in both groups. As “group A” vs “group B” regarding breast self-examination, n=13 (16.7%) vs n=10 (31.3%); clinical breast examination n=4 (5.1%) vs n=4 (12.5%) and mammography was not performed by any woman in both groups.

**Conclusion:**

the modest knowledge and poor BCS practices of our study groups strongly recommends appropriate official and educational actions.

## Introduction

Breast Cancer (BC) has the highest incidence and is the first cause of death from cancer in women worldwide. Despite extensive Breast Cancer Screening (BCS) programs in the western countries, BC still causes significant morbidity. Currently, the estimated number of newly diagnosed BC cases worldwide is about 2.1 million [1]. Hence, BC is recognized as a major public health problem in both developed and developing populations and its cost increases with the advancement of the disease stage at diagnosis [2]. In comparison, African women despite their lower incidence of cancer, are burdened with higher cancer mortality rates when compared to the western women [1,3,4]. Awareness and attitude concerning BC screening were described as common factors that determine the stage at which patients with BC present to the hospital. Consequently, low BC awareness added to lack of screening and control programs made many African women being diagnosed late [5]. On the other hand, it is well known that BC awareness and screening assisted detection of BC at an early stage, and improved their outcome [4,6].

Sudan as a large developing African country is struggling to provide essential preventive health services in low resource settings. The few available records showed that Sudan is experiencing a rapidly increasing BC prevalence and high incidence characterized by late and complicated stages at presentation, and a high frequency related to a several risk factors [7]. Additionally, BC in Sudanese women happens at younger age compared to western women as about 40% of the patients are under the age of 45 years. The fact that the majority of the patients present at late disease stages made BC to be the leading cause of cancer related death in the Sudan [1,8]. The perception of BC screening and its different methods such as regular Breast Self-Examination (BSE); routine Clinical Breast Examination (CBE) and mammography are believed to be lacking at a large scale in our community [9]. The important role of early detection as the main protection against consequent serious sequelae can`t be missed. Upon the shoulders of health caregivers, especially females this burden is put. Their individual adequate awareness, attitude and practice is the main weapon to win this challenge. Moreover, studies in the Sudan addressing this issue among health care providers are so scarce. The objective were to carry out the study to assess and compare the healthcare providers` level of knowledge attitude and practice concerning BCS.

## Methods

**Design:** a descriptive, hospital-based cross-sectional study.

**Study setting:** study was conducted during the period from November 2016 to January 2018 at Ad-Damazin Teaching Hospital (containing 271 beds and 483 employees and several clinical specialities). This is the main governmental state hospital serving over 1100000 papulation in Ad-Damazin City, capital of the Blue Nile State in the South-Eastern Sudan. Hence, it can be defined as a secondary-care institution [10].

**Participants:** the study population were adult female university graduates and postgraduates working at that hospital. The medical personnel comprised doctors, pharmacists, nurses, radiology technologists, laboratory scientist and technologists, dieticians. The non-medical participants were the accountants, statisticians and other office employees.

**Exclusion criteria:** women who suffered from BC or any other cancer; women in a holiday and women been absent for any reason during data collection period were not included in this study.

**Variables:** included in 97 items within four sections: Section 1: sociodemographic data included age; marital status; occupation; level of education; work experience; health insurance status and having close person suffered from breast cancer (9 items). Section 2: knowledge (21 items) divided into predisposing factors; clinical manifestations and BCS methods (BSE, CBE and mammography). Section 3:attitude assessment based upon the revised Champion's Health Belief Model Scale (CHBMS) six concepts and 63 items processes [11,12]. The concepts included susceptibility; severity; benefits; barriers; self-confidence and health motivation (Details in Supplementary file, 1)[11-13]. Section 4: practice regarding early detection methods of BC (BSE; CBE and mammography) (3) items.

**Data collection technique:** through direct interview of the participants by the principle researcher.

**Data collection tool:** was a predesigned, pretested and a self-administered questionnaire. Its validation was via a draft questionnaire designed after meticulous search in the literature. Its contents were face validated and reviewed with expert consultants. A pilot test was carried out in 20 participants (10 medical and 10 non-medical, not included in the main study) working at Sudanese Chinese Friendship Hospital at Ad-Damazin City. The pre-test data was analysed and the final questionnaire was produced after elimination and modification of some items.

**Questionnaire reliability:** Alpha Cronbach´s test was performed to assess the internal consistency and reliability of the questionnaire items. A high value of the test coefficient (0.939) was obtained. Response rate 94.8 % (110/116 participants, as 6 participants did not fill the questionnaire). The average time to answer this questionnaire was 12-16 minutes.

**Questionnaire format, scoring and grading system:**

**Knowledge:** the questions were close-ended of the “Yes”, “No” or “I don´t know” format. For each correct answer a candidate was awarded one mark and a zero mark for wrong, “I don´t know”, blank or a more than one checked answers. A full mark was 21, graded as “Poor” (0-7 (≤ 33%)); “Moderate” (8-14 (34-65%)) and a “Good” (15-21 (≥ 66%)) level of knowledge.

**Attitude:** assessment format was three-point Likert scale (including the options, I agree; I´m neutral and I don´t agree) was used to a cover narrow range of attitude. For every positive attitude “I agree”; the participant was awarded one mark, for negative attitude “I´m neutral” or “I don´t agree” answer the participant was awarded zero mark. A full attitude score was 63 points, graded as “Negative” = 0-31 (< 50%) and “Positive” ?32 (?50%) level of attitude.

**Practice:** assessment format was “Done” and “Not done”. For every best practice screening methods the participant was awarded one mark for “Done” response, and for “Not done” response, zero mark was awarded. Highest practice score was three marks, where score of 0-1 graded as unsatisfactory and 2-3 as satisfactory level of practice. To identify the medical education effect, the participants were divided into medical personnel “group A”, and non-medical participants “group B” for comparison purposes.

**Potential bias management**: selection bias: as a cross sectional study the potential bias due to convenience sampling was eliminated by the high response rate (94.8 %) of the participants. Information bias: The information bias was addressed by using a the valid and reliable Champion's Health Belief Model Scale.

**Confounding:** to avoid confounding causal comparison was avoided in this study.

**Sampling:** addressing selection bias a nonprobability (convenience) sampling of all the females working at Ad-Damazin Teaching Hospital and satisfying the inclusion criteria was approved.

**Statistical analysis:** data completeness assessment, cleaning, entry, then analysis via Statistical Package for Social Sciences (SPSS ver. 16, IBM Corp., USA) was accomplished. Regarding knowledge, attitude and practice among the two groups´ percentage and mean scores of each piece of data calculated and analysed, excluding missing data. Categorical variables were noted in numbers and percentages. Continuous variables were expressed as mean ± standard deviation. Statistical methods used were Chi-squared tests for association, independent t-test to compare the both groups for each mean of independent variables, the p value of significance was < 0.05.

**Ethical issues:** ethical approval for this study was obtained from the Dean Faculty of Medicine, Blue Nile University ethical committee and the General Secretary for Ministry of Health. The participants were informed and consented for the study. Participants` confidential information was guaranteed through unnamed questionnaire. This study assessed the level of BCS among Sudanese female workers at a secondary healthcare hospital.

## Results

**Socio-demographic:** the study included 110 (78, medical staff and 32, non-medical staff) participants. The majority of the participants in both groups, were young (22-39 years); married; nurses and technicians. Most of them had less than five years´ experience and have health insurance. A minority of them knew some person who had cancer before. Details of their socio-demographic data are shown in (Table 1).

**Table 1:** socio-demographic data of the participants, comparing medical group versus non-medical group, n=110

Socio-demographic items	Medical group, n=78		Non-medical group, n=32		P value
	n	%	n	%	
**Age in years:**					
22-39	74	94.9	30	93.8	
40 or more	4	5.1	2	6.2	0.281
**Marital status**					
Married	39	50	16	50	
Single	36	46.2	15	46.9	0.932
Divorced, Separate, Widow	3	3.8	1	3.1	
**Total**	78	100	32	100	
**Occupation:**					
Doctors and pharmacists	22	28.3			
Nurses and technicians	36	46.1			> 0.001*
Other medical professions	20	25.6			
Non-medical professions			32	100	
**Total**	**78**	**100**	**32**	**100**	
**Education**					
Graduate	72	92.3	30	93.8	0.791
Postgraduate	6	7.7	2	6.2	
**Total**	**78**	**100**	**32**	**100**	
**Work experience**					
Less than five years	41	52.6	19	59.4	0.515
Five years or more	37	47.4	13	40.6	
**Total**	**78**	**100**	**32**	**100**	
**Included in health insurance**					
Yes	65	83.3	30	93.8	0.148
No	13	16.7	2	6.2	
**Total**	**78**	**100**	**32**	**100**	
**Do you know someone suffer from breast cancer**					
No	55	70.5	22	68.8	0.545
Yes	23	29.5	10	31.2	
**Total**	**78**	**100**	**32**	**100**	

(*) means significant p value

**Missing data:** none.

**Outcome measures:** are knowledge, attitude and practice of both groups towards BCS.

**Knowledge:** comparing “group A” vs “group B”, the mean score of knowledge regarding of the predisposing factors were 4.96 ± (2.4) vs 3.19 ± (2.7), p=0.001; clinical manifestations were 5.88 ± (2.5) vs 4.62 ± (2.5), p=0.019; early detection methods were 1.91 ± (1.1) vs 1.34 ± (1.2), p=0.020. The highest overall knowledge grade was “moderate” (60.8% for “group A” and 43.6% for “group B”), p=0.000.

**Figure 1:**
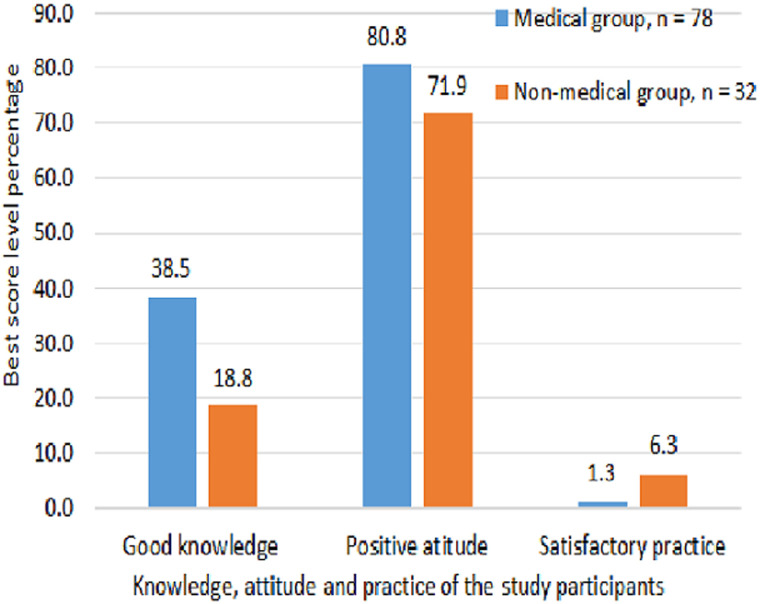
breast cancer screening factors´ best scores among Ad-Damazin Hospital female workers, n = 110

**Attitude:** positive attitude score was detected in the majority of both groups, as demonstrated in (Table 2, Figure 1).

**Table 2:** scores of attitude and grades of various components of breast cancer screening among the participants´; medical group Vs non-medical group; n=11

Breast cancer Item (Item score)	Medical group, n=78		Non-medical group, n=32		P value
	Mean ± (SD)	%	Mean±(SD)	%	
Susceptibility of having breast cancer (4 points)	0.27±(0.8)	6.8	0.41±(0.9)	10.3	0.409
Severity of breast cancer (6 points)	3.46±(1.4)	57.7	3.03±(1.4)	50.5	0.141
Health motivation of breast cancer awareness and early detection methods (3 points)	2.82 ± (0.7)	94	2.75±(0.7)	91.7	0.621
Confidence in breast self-examination performance (8 points)	5.41± (2.9)	67.6	5.28±(2.4)	66	0.822
Benefit of breast self-examination (6 points)	5.24±(1.5)	87.3	4.81±(1.6)	80.2	0.181
Barriers regarding breast self-examination (5 points)	3.88±(1.5)	77.6	3.94 ± (1.4)	78.8	0.867
Benefits of clinical breast examination (6 points)	5.10±(1.5)	85	5.03± (1.8)	83.8	0.829
Barriers to attend clinical breast examination (10 points)	5±(3.2)	50	5.44±(3.3)	54.4	0.523
Benefit of mammography (6 points)	4.04±(2.4)	67.3	3.16±(2.2)	52.7	0.072
Barriers to mammography (9 points)	3.33±(2.9)	37	3.06±(2.9)	34	0.655
**Total score (63)**	38.56 ±(12)	61.2	36.91 (±10.5)	58.6	0.498
**Score grade association**	**n**	**%**	**n**	**%**	**P value**
Good knowledge and positive attitude	n=20	25.6	n=3	9.4	0.002*
Good knowledge and satisfactory practice	n=1	1.3	n=0	0	0.469
Positive attitude and satisfactory practice	n=1	1.3	n=2	6.3	0.354

SD = Standard deviation; (*) means significant p value

**Practice:** satisfactory BCS practices regarding BSE were reported in 13 (16.7%) participants in “group A” and in ten (31.3%) participants in “group B”, p=0.045. Satisfactory CBE was reported by four (5.1%) participants in “group A” and by four (12.5%) participants in “group B”, p=0.039 where mammography was not performed by any participant, more details shown in (Figure 1). Mean score of knowledge, attitude and practice regarding breast cancer screening among the different sectors of the health care providers showed little differences (Figure 1). Comparisons of knowledge, attitude and practice of the participants´ scores and grades, for the participants scoring combined “good knowledge and positive attitude” the score of both groups was low and the medical participants were significantly better than the non-medical participants. When “satisfactory practice” come in combination with “good knowledge” and “positive attitude” the score was low and the medical personnel were not better than non-medical participants. Details are shown in (Table 2).

## Discussion

**Sociodemographic characteristics:** our study assessed the graduate females working at the largest hospital of Ad-Damazin City (Table 1). They are expected to have a higher sensitivity towards breast cancer because they are highly-educated and most cases of breast cancer in the region are referred to their hospital. Since the medical staff represented the largest group, n=78 (70.9%), we believe that our overall results of knowledge, attitude and practice to a large extent represent that group of healthcare providers. The age group of 22-39 years was the majority (94.9%) of our combined sample. It has been found that over 33% of African breast cancer women ages were 30-49 years, and 81% age ranged between 30-59 years [14].

Our larger young age group represents a good chance to assess the situation at this age group who can really benefit from early detection of breast cancer. Although half of our participants are married, early discovery of breast cancer and the role it plays in the prevention of the marriage dissolution and lowering the risk of divorce is controversial [15,16]. Most of our “group A” participants were nurses and technicians, n = 36 (46.1%), this finding may suggest for health planner the sector of health care workers to pay more attention in training to play important roles in instituting screening strategies for breast cancer. The majority of both groups are included in the health insurance services. Antabe *et al*. have demonstrated that there is a positive association between the breast cancer screening and including in health insurance service [17]. In almost one third of each group of our surveyed participants we found that someone near to them suffered from breast cancer (29.5% of group A and 31.3% of group B). Those participants were supposed to have a better knowledge, positive attitude and adequate practice, this fact proved by some previous studies [18,19].

**Knowledge:** the assessed knowledge components regarding (risk factors; clinical manifestations; and early detection methods) revealed that “group A” was significantly better than “group B”. The highest score grade of overall knowledge in “group A” participants was moderate, while that of “group B” was poor. The poor knowledge outcome detected in our “group B” go on the same line with earlier surveys carried out regionally in Nigeria and Yemen [20,21]. We believe that lack of health educational programs concerning BC for the “group B” at the education or community level may explain the poor level of knowledge. Where, the relatively modest knowledge score of the “group A” working in secondary level health care institution is difficult to explain.

**Attitude:** assessment of attitude in both groups revealed their almost equal positive trends towards certain elements of attitude regarding breast cancer screening (Table 2). They described BC as markedly sever with serious effects on their lives; their high motivation to care for their own health and the fact that the screening measures (breast self-examination, clinical breast examination and mammography) are beneficial without major obstacles to perform them. Specifically, a negative attitude and denial trend towards susceptibility to developing BC was stated by the participants of both groups. The reason for this negative belief could not unfortunately, be detected by this study. The same table shows lack of access to screening mammography expressed by the majority of the participants.

Although the mammography machine is not really expensive, most of our radiology departments lack it while they are equipped with Computerized Tomography (CT) and Magnetic Resonance Imaging (MRI) machines. The reason for this defect is unknown and reflects a defective public and medical authority strategy towards BCS in our country. A moderate (unclear and unsatisfactory) level of confidence to perform breast self-examination was also detected by our survey. The overall attitude in score (Figure 1) presented in our study was positive in both groups with relatively higher magnitude in the medical group versus non-medical. These findings are in accordance with similar other studies conducted in many countries such as Iran and Jordan [22,23]. This positive attitude trend may reflect a global situation of attitude regarding the BCS despite expected cultural variation among the various communities. Unfortunately, the similarity or lack of significant superiority of the medical group in many attitude items is somewhat worrying.

**Practice:** concerning early BCS, knowledge and attitude-directed practice had already been emphasized [13,24]. On the other hand, it is well known that even in graduated women with insufficient BC screening-focused knowledge and attitude, good practice is not expected [25]. Concerning our participants, their practice components scores for BSE were less than a fifth of our medical participants (16.7%) and almost one third (31.3%) of the non-medical group reported monthly performing BSE with superiority of the non-medical group. Although this result goes in line with the studies carried by Ahmed *et al*. and Bener *et al*. [26,27]. This fact contradicts previous studies conducted in Sudan in 2013 by Idris and associates, which showed more than half of their female medical staff participants were performing BSE monthly [28]. This fact raises the question that do medical students behave different after graduation with regard to BC screening? A disappointingly, very poor practice rate of clinical breast examination (CBE) was noted in both groups (5.1% vs 12.5%) with even a relatively higher practice rate in the “group B”.

**Figure 2:**
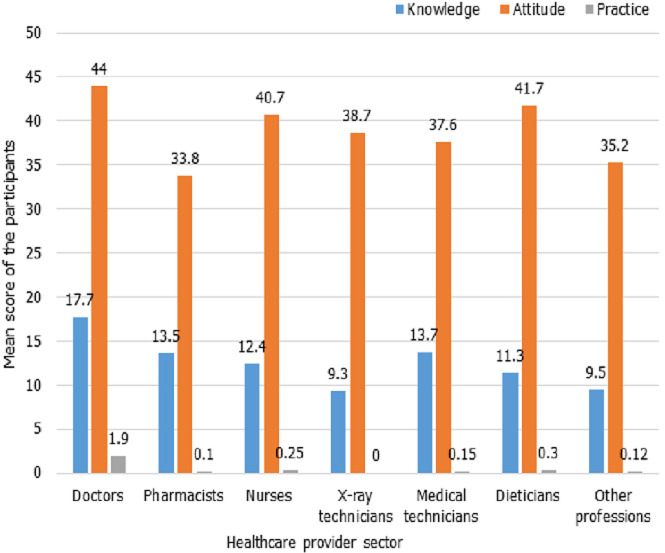
knowledge, attitude and practice regarding breast cancer screening mean score distribution among Ad-Damazin Hospital female medical workers, n=78

Our study finding that healthcare workers are not doing any better regarding BCS practice, regardless of the knowledge and attitude detected levels (Figure 1). Inadequate practice of healthcare workers had been reported by many researchers before, regionally and globally such as United Arab Emirates, Tunisia, India and USA [29-31]. Our study may not point out the real cause for poor BCS practice phenomenon. A possible explanation may be that medical schools teach students well about BC and “breast examination” without (maybe) equal emphasise and training on “breast self-examination”. The sensitivity of the issue may explain that. Moreover, medical curricula prepare students to examine patients, yet not to examine themselves. A slightly different regional study from Saudi Arabia assessing BCS included 395 healthcare workers detected reasonable knowledge and BSE practice, but extremely poor practice levels of CBE and mammography [32]. Our study revealed these poor results despite the fact that CBE is currently considered as a very effective alternative screening method for BC in developing regions; when other screening modalities are not cost-effective and not available at public base [33]. Regarding mammography, in both study groups no participant reported being screened by mammography due to nonexistence of nearby mammography services. A similar result was encountered in Uganda because mammography wasn`t used as a screening method [34]. Saudi researchers in 2014, reported zero participation rate in mammography among their surveyed ladies despite the accessible mammogram services, for unrecognized reason [35]. The overall outcome of practice in both groups is very poor (1.3% vs. 6.3%) in “group A” versus “group B”. The mean score of knowledge, attitude and practice regarding BCS among the different sectors of the health care providers showed little differences (Figure 2). This finding may indicate a basic lack of this issue in the medical education of almost all of the health providers.

Table 2, regarding grades, reveals significant association between good knowledge and positive attitude, p=0.002, no association between good knowledge and satisfactory practice, p=0.469 and no association between positive attitude and satisfactory practice, p=0.354. This is a very perplexing finding. We think that lack of harmony among these three items, as demonstrated above may be due to the priority setting of the health care authorities in our developing country. Because of its limited recourses it may direct most attention towards communicable diseases on expense of the non-communicable diseases. Similar results were obtained in Nigeria by Nanloh S Jimam *et al*.[36]. An added possibility may be the participants´ denial to being susceptible to BC. That belief makes them less likely to peruse health preventive behavior. Many similar studies established the fact that knowledge, attitude and practice may go inconsistently. Studies performed in 2015 among Saudi medical students and Malaysian school teachers detected similar discord between these three items [37,38]. Our study proves that the BCS susceptibility perception and practical education, as well is seriously defective.

Our study limitations were that it was a single center; hospital-based (rather than community-based). Assessment of practice was so brief and in a form of close-ended questionnaire, rather than actual observation due to the practical sensitivity of the issue. Moreover, undergraduate and community-based ladies were outside the scope of this study. The results of our study open the door widely for further studies to detect reasons behind the negative attitude towards BC susceptibility; lack of mammography machines in most of our hospitals; the lower confidence level to perform breast self-examination by our participants and the inferiority of the medical group compared to non-medical group in performing breast self-examination.

## Conclusion

Concerning BCS, our study of medical and non-medical staff showed obvious denial features towards susceptibility to a very well-known threatening disease. Furthermore, the study highlighted the alarming poor practice accompanying moderate knowledge and positive behavior. These findings noticeably necessitate further generalization via national studies and in-depth investigation of the possible local socio-cultural and educational causes. Doctors and rest of health care providers should highly be focused as leaders of the health care services leading other woman along the road to successful BCS practice.

### What is known about this topic

- *Advanced cases of breast cancer are seen in Africa;*
- *Breast cancer screening helps in early detection of breast cancer*.

### What this study adds

- *The level of knowledge, attitude and behavior regarding breast cancer screening among healthcare providers in a big city in the Sudan is seriously low;*
- *The knowledge, attitude and behavior level regarding breast cancer screening among those healthcare workers is not better than the ordinary (non-medical) workers*.
